# Enterprise Characteristics Were Associated With Adherence to a Dialog-based Inspection Practice Aimed at Improving Occupational Safety and Health in Denmark

**DOI:** 10.1016/j.shaw.2025.05.002

**Published:** 2025-05-11

**Authors:** Mikala E. Jakobsen, Asta Kjærgaard, Emilie M. Rudolf, Julie Palmqvist, Jeppe Z.N. Ajslev

**Affiliations:** The National Research Centre for the Working Environment, Copenhagen, Denmark

**Keywords:** Enterprises, Inspection, Prevention, Safety, Work

## Abstract

**Background:**

Risks in the psychosocial and ergonomic working environment can be complex and difficult for labor inspectors to uncover. In 2020, authorities implemented a dialog-based inspection practice in order to improve the working environment in all types of Danish enterprises.

**Methods:**

The study sample consisted of 3183 enterprises and stemmed from a quasi-experiment, Agreement To Problem-solve (ATP), implemented nation-wide by Danish authorities. Enterprises that were offered an ATP could accept this voluntary agreement if the labor inspectors suspected a complex health and safety problem, especially problems in the psychosocial or ergonomic working environment. We used univariate and multivariate logistic regression models to investigate associations between enterprise characteristics and fulfillment of the ATP.

**Results:**

In total, 2335 enterprises fulfilled an ATP from 2020 to 2023. Compared to *Public administration, education and health care*, the industries *Industrial, raw materials and supply* and *Trade, transportation etc.* had significantly lower odds ratios (ORs) of fulfilling the agreement [OR: 0.70, 95% confidence interval (CI): 0.53; 0.92 and OR: 0.6, 95% CI: 0.46; 0.80, respectively]. Enterprises with more than 35 employees had a significantly higher probability of fulfilling the agreement than enterprises with 1–9 employees. The OR of fulfillment regarding ergonomics was 0.75 times that of the psychosocial (95% CI: 0.58; 0.98).

**Conclusion:**

This dialog-based inspection practice showed promising results in regards to addressing and terminating suspicions regarding the psychosocial and ergonomic working environment. The enterprises' industry, number of employees, and type of occupation safety and health problem were associated with fulfillment of this dialog-based inspection practice.

## 1 Introduction

As the society develops, the need for diverse initiatives regarding occupational safety and health (OSH) increases. This is in order to prevent risks and adverse outcomes in the working environment effectively. Specifically, risks associated with illness or injuries over time are often difficult to mitigate, and effective enforcement is difficult to achieve for authorities due to the complexity of these OSH risks. The psychosocial and ergonomic working environment consists of issues that are complex, are difficult to uncover, and are specifically associated with long-term sickness absence [[1], [2], [3]].

New ways of working, a changing labor market, and new technologies bring new challenges and OSH risks in the working environment, which in turn require extended skill sets and competences in the field of labor inspection [4].

Inspectors face dilemmas in the meeting with workplaces as new tasks demand a change from traditional methods of direct control and advice to negotiation, guiding, and tutoring [5].The Working Environment Authorities (WEAs) in Scandinavia are characterized by a double role of controlling compliance with the law and guiding enterprises regarding OSH and initiatives [6,7].

Some hope has, however, been set to inspection activities based on dialogue between employers and labor inspectors as well as combinations of communication and information as these have shown positive outcomes regarding psychosocial risks [8].

A dialogue-based (sometimes called participatory or co-operative) inspection is one where the labor/OSH inspector deliberately devotes time to a two-way conversation with both management and workers. The visit focuses on diagnosing hazards and causes and agreeing on realistic improvements rather than just issuing sanctions, thus motivating the enterprise to continue the work after the inspector leaves. Dialog-based methods show the strongest effects in high-risk or resource-limited settings where practical know-how resides with frontline workers [9].Another study found positive outcomes of inspectors' interventions on psychosocial risks in supportive contexts and with appropriate training and resources [8].

A two-way communication and dialog-based inspection approach has shown satisfactory results for both inspectors and enterprises [10,11].

Knowledge on how authorities may effectively regulate and intervene on psychosocial and ergonomic risks of the working environment is however scarce, and a review from 2019 identified a “major research gap” regarding the effects of OSH regulations targeting psychological and musculoskeletal disorders [7].

Weissbrodt et al also stated that strong evidence is lacking and that more evaluation studies are necessary [8].

In order to reap the benefits of dialog-based approaches, Danish authorities implemented a new dialog-based reaction form in 2020, the Agreement to Problem-solve (ATP). The ATP should address and resolve issues with psychosocial and ergonomic OSH risks in enterprises. Psychosocial and ergonomic risks generelly receive few injunctions from the WEAs compared to other OSH risks [13]. Thus, the ATP aims to solve OSH problems regarding the psychosocial and the ergonomic working environment. This means that under certain conditions, enterprises could choose a dialog- and-guidance-based intervention instead of receiving an injunction when the labor inspector suspected an OSH problem or risk.

The aim of implementing ATP was to save time uncovering these complex OSH problems and at the same time offer the enterprises autonomy and opportunity to uncover and implement appropriate solutions with guidance from a labor inspector.

In order for the ATP to be a successful way for authorities to address psychosocial and ergonomic OSH issues, it was pivotal that the enterprises completed the ATPs. As such, it is highly important to investigate whether companies actually fulfilled the ATPs.

In addition to this, it is highly interesting to investigate the fulfillment of ATPs in different types of enterprises. This is, for instance, because small- and medium-sized enterprises have barriers to OSH interventions in the sense that they often have limited resources to prioritize and improve the working environment compared to large enterprises [[14], [15], [16]]. In addition, studies on the significance of sector and type of industry regarding OSH interventions are scarce.

There is little knowledge on what type of enterprises, if any, are more susceptible to and benefit from this sort of modern inspection practice that focuses more on dialog rather than injunction. More research on larger enterprises and enterprises in different sectors and with different types of jobs is needed in order to understand possible underlying mechanisms and to target different kinds of inspection practices to different kinds of enterprises as to improve the working environment as much and effectively as possible.

To our knowledge, this is the first study to investigate the association between various enterprise characteristics and the compliance with this type of dialog-based inspection practice.

The aim of this study was to investigate the association between enterprise characteristics and the fulfillment of the ATP intervention targeting psychosocial and ergonomic risks in the working environment. The purpose of our study was to contribute to the research field regarding this type of dialog-based labor inspection practices and their potential to improve and reduce risks in the working environment for various types of enterprises.

## 2 Materials and methods

### 2.1 Study design and participants

We analyzed data from a quasi-experiment implemented nation-wide by the Danish WEA in 2020; ATP, and derived from a national evaluation of the ATP conducted for the WEA in 2024.

Since 2020, the WEA has been able to offer enterprises a voluntary ATP if the labor inspectors suspected a complex OSH problem, especially problems in the psychosocial or ergonomic working environment. The ATP provides enterprises the opportunity to implement their own initiatives regarding the working environment by solving a health and safety problem while receiving additional help and guidance from the WEA during the process.

When offered an ATP regarding complex problems, the enterprises agree to develop and implement a solution to their OSH problem, and in return, the WEA will stop investigating the potential violation of the substantive rules of the Working Environment Act. The WEA does not offer ATP for problems that need to be solved immediately, are immanently dangerous, or require other severe types of reactions. The enterprises needed to solve the OSH problem within a time limit, and at the end of this, the labor inspector conducts a final inspection and decides whether the enterprise has fulfilled the ATP or not [17].

In total, the WEA offered an ATP to 4129 enterprises from 2020 to 2023. ATPs that 1) had been offered but not yet accepted or declined and 2) ongoing ATPs were excluded from analyses (N = 946) resulting in a study sample of 3183 enterprises. We included ATPs that were fulfilled, not fulfilled, rejected, or interrupted in the analyses. We gave the ATP the status “Not fulfilled ATP” if the ATP was not fulfilled, rejected, or interrupted and the status “Fulfilled ATP” if the ATP was fulfilled.

The WEA made data available to us, which had information regarding the enterprises' registered industry, the number of employees, type of OSH problem, fulfillment status, year of offer, sector (public/private), and geographical placement of the working authority supervision center responsible for the ATP.

We allocated the enterprises to one of 10 industry categories [18]. We pooled the industries with less than 50 observations in an “Other” category. The industries included in the “Other” category were *Agriculture, forestry and fishing* (n = 35), *Construction* (n = 8), and *Information and communication* (n = 31).

### 2.2 Statistical analyses

Descriptive analyses used the chi-square test. We identified associations between industry, number of employees and type of OSH problem and fulfillment-status using univariate and multivariate logistic regression models. We adjusted the final model for potential confounders: year of offer and supervision center. Estimates are presented as odds ratio (OR) and with a 95% confidence intervals (95% CIs).

We used the software SAS 9.4 (SAS Institute Inc.) for data management and analysis.

## 3 Results

In total, 2335 enterprises (73%) fulfilled an ATP, and 848 (27%) did not fulfill, declined, or interrupted the ATP (Table 1).

**Table 1:** Enterprise characteristics and status of Agreement to Problem-solve (ATP)

	Not fulfilled ATP	Fulfilled ATP	Total *n*
**Industry**			
Public administration, education, and health care	269 (20)	1051 (80)	1320
Culture, leisure, and other services	42 (29)	101 (71)	143
Industrial, raw materials, and supply	180 (28)	462 (72)	642
Office	44 (20)	177 (80)	221
Trade, transportation, etc.	294 (38)	489 (62)	783
Other	19 (26)	55 (74)	74
**Number of employees**∗			
1–9	111 (33)	222 (67)	333
10–34	282 (32)	586 (68)	868
35–99	191 (21)	723 (79)	914
+100	154 (18)	689 (82)	843
**Type of OSH problem**			
Psychosocial	208 (18)	941 (82)	1149
Noise	97 (33)	196 (67)	293
Ergonomics	344 (32)	721 (68)	1065
Chemistry, dust, and biology	76 (27)	203 (73)	279
Indoor climate	92 (37)	160 (63)	252
Accident risk	26 (20)	101 (80)	127
Other	5 (28)	13 (72)	18
**Sector**			
Private	559 (32)	1209 (68)	1768
Public	255 (21)	961 (79)	1216
Other	34 (17)	165 (83)	199
**Supervision center**			
Northern	187 (25)	548 (75)	735
Southern	252 (27)	697 (73)	949
Eastern	409 (27)	1090 (73)	1499
**Year of offer**			
2020	84 (27)	230 (73)	314
2021	279 (24)	901 (76)	1180
2022	233 (21)	881 (79)	1114
2023	252 (44)	323 (56)	575

Data are given as number (*N*) and percentage (%).

OSH, occupational safety and health.

∗ There are 225 enterprises with an unknown number of employees.

*Public administration, education and health care*, *Trade, transportation etc.* and *Industrial, raw materials and supply* were the industries with most ATPs. *Public administration, education and health care* had nearly twice as many ATPs as *Trade, transportation etc*. *Culture, leisure and other services* had the least ATPs, apart from the “Other” pool of industries.

Problems regarding the psychosocial and ergonomic OSH made up the majority of the ATPs, which aligns with the WEA's purpose with the reaction form.

Table 1 shows that specific industries were represented more than others in the ATPs. Industries with the largest proportion of fulfilled ATPs were *Public administration, education, health care*, and *Office*.

Enterprises with 35 or more employees had larger proportions of fulfilled ATPs (about 80%) than smaller enterprises (70%). Larger enterprises had more ATPs overall.

There were more ATPs regarding psychosocial and ergonomic OSH than other OSH problems, but the psychosocial ATPs had a larger proportion of fulfillment than ergonomics with 82% and 68%, respectively.

More enterprises within the private sector had accepted an ATP than within the public sector, but the public sector had a greater proportion of fulfillment (79%) than the private sector (68%).

The supervision center primarily covering Eastern Denmark had the most ATPs, although the proportion of fulfilled ATPs was the same across centers. There were fewer ATPs in 2020 since it was the first year of the intervention and it might have required some time and adaption to be fully integrated in the WEA's catalog of reactions. Likewise, there were fewer fulfilled ATPs in 2023 than in 2021 and 2022 because some of these were still ongoing when the analyses were conducted.

**Fig. 1:**
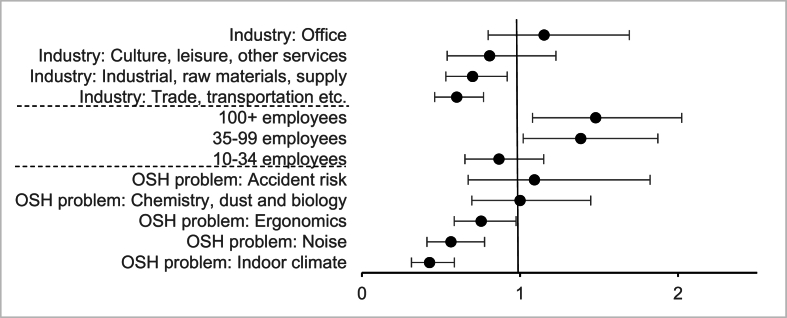
Odds ratios and 95% confidence intervals of enterprises' fulfillment of the Agreement to Problem-solve (ATP). Adjusted for year of offer and supervision center. Industry reference = Public administration, education, healthcare; number of employees reference = 1–9; OSH problem reference = psychosocial. OSH, occupational safety and health.

Sector correlated with industry, the number of employees, and OSH problems. In a model including the four independent variables, sector showed no association with the fulfillment of ATP. Therefore, we left sector out of the final and adjusted model represented in Table 2 and Fig. 1.

**Table 2:** Adjusted model of enterprise characteristics, type of OSH problem and Agreement to Problem-solve (ATP) fulfillment status

	Fulfilled ATP	95% CI	*p*
	Odds ratio		
**Industry**			
Public administration, education, and health care	Ref. (1)		
Culture, leisure, and other services	0.81	[0.54; 1.23]	0.3070
Industrial, raw materials, and supply	0.70	[0.53; 0.92]	**0.0103**
Office	1.15	[0.80; 1.69]	0.4592
Trade, transportation, etc.	0.60	[0.46; 0.80]	**<.0001∗**
**Number of employees**			
1–9	Ref. (1)		
10–34	0.87	[0.65; 1.15]	0.3251
35–99	1.38	[1.02; 1.87]	**0.0360**
+100	1.48	[1.08; 2.02]	**0.0147**
**Type of OSH problem**			
Psychosocial	Ref. (1)		
Noise	0.56	[0.41; 0.78]	**0.0004**
Ergonomics	0.75	[0.58; 0.98]	**0.0311**
Chemistry, dust and biology	1.00	[0.70; 1.45]	0.9998
Indoor climate	0.43	[0.31; 0.59]	**<.0001∗**
Accident risk	1.10	[0.67; 1.82]	0.7297

Bold data refer to statistically significant results (*p* < 0.05).

∗: *p* < 0.001. Adjusted for year of offer and supervision center.

CI, confidence interval; OSH, occupational safety and health.

Table 2 shows that compared to *Public administration, education, health care* the industries *Industrial, raw materials, supply* and *Trade, transportation etc.* had significantly lower ORs of fulfilling the ATP (OR: 0.70, 95% CI: 0.53; 0.92 and OR: 0.6, 95% CI: 0.46; 0.80, respectively).

Enterprises with 35–99 and 100+ employees had a significantly higher probability of fulfilling the ATP with ORs of 1.4 and 1.6, respectively, compared to enterprises with 1–9 employees.

The OR of fulfillment regarding ergonomics was .75 times that of the psychosocial (95% CI: 0.58; 0.98). Indoor climate and noise OSH problems were significantly associated with a lower probability of fulfillment than psychosocial OSH problems with ORs of 0.44 and 0.55, respectively (Table 2).

Model testing showed a good fit with *p* values smaller than 0.0001 regarding score, likelihood ratio, and Wald test. Somers’ D was 0.37.

Supervision center was not associated with fulfillment in the adjusted model, and removing this from the model did not change its fit.

### 3.1 Sensitivity and supplementary analyses

A univariate regression showed that public enterprises had a greater probability of fulfilling ATP (OR = 1.7) than private enterprises. Furthermore, the majority of public enterprises consisted of 35 or more employees, and the majority of private enterprises consisted of 1–34 employees. In addition, private enterprises mainly received ergonomic-related ATPs and public enterprises mainly received psychosocial ATPs.

In the four-year period from 2020–2023, 768 ATP offers were rejected or interrupted. An analysis showed that psychosocial ATPs were rejected or interrupted the least offers with 11% of offers and that ergonomic ATPs were rejected or interrupted 25%. Noise ATPs were rejected or interrupted 22%.

In enterprises with more than 100 employees, 10% rejected or interrupted the ATP. In 35- to 99-employee enterprises, it was 14%; for 10- to 34-employee enterprises, it was 25%; and for 1- to 9-employee enterprises, it was 24% that rejected or interrupted the ATP. This coincides with 35- to 99- and 100+-employee enterprises having greater ORs than 1- to 9-employee enterprises of fulfilling the ATP.

Analyses including more detailed categorization of industries showed similar results to those represented in Table 2; industries included in the *Trade* and *Industrial* categories such as *Restaurants*, *Shops* and *Slaughterhouses* were associated with less fulfillment of ATP than *Residential institutions and home care* (categorized as *Public administration, education, and health care*).

In an adjusted regression model including only the enterprises in *Public administration, education and health care*, ATPs regarding noise was no longer adversely associated with fulfillment of ATP. Indoor climate (OR = 0.3) and ergonomics (OR = 0.6) maintained smaller ORs of fulfilling ATP than psychosocial ATPs. In addition, 66% of ATPs in *Public administration, education and health care* regarded psychosocial problems. In this stratified analysis, the number of employees was no longer associated with fulfillment, and the 1- to 9-employee group had no more than 40 observations. The 35- to 99- and 100+-employee groups had 487 and 425 observations, respectively.

## 4 Discussion

The largest industry group was *Public administration, education and health care* and represented 41% of all enterprises. Enterprises working in *Trade, transportation, etc*. and *Industrial, raw materials and supply* had a lower probability of fulfilling an ATP than *Public administration, education and health care*. Enterprises with more than 34 employees had a higher probability of fulfillment than small enterprises, and OSH problems regarding noise, ergonomics, and indoor climate had lower probability of fulfilling ATP than psychosocial OSH problems.

The results indicate that small enterprises have a lower probability of fulfilling the ATP than bigger ones, which coincides with previous studies that identified various barriers for solving OSH issues in small enterprises [19]. Large—and often public—enterprises might generally have greater resources to work with the ATP and OSH problems, and in Denmark, only enterprises larger than those with 10 employees are obligated to establish OSH organizations within the enterprises [20]. The ATP often entails that the enterprise conducts its own investigation and defines the problem and the solution, which can be time-consuming and costly, especially for small enterprises without established internal OSH organizations [19]. However, the ATP experienced high fulfillment (70%), even in small enterprises. While there are limitations to the approach, it seems highly promising as a means for addressing OSH in small enterprises as well as large ones.

ATPs regarding chemistry, dust, and biology OSH problems were not associated with fulfillment of ATP, while the industry *Industrial, raw materials, supply* was. The correlation between these types of OSH risks and the industry might explain the missing association between chemistry, dust, and biology and fulfillment of ATP.

Enterprises with 10–34 employees did not show a significant association with fulfillment. This could be because small enterprises more often than large ones were offered ergonomic ATPs and that small enterprises are primarily found in the *Trade* or *Industrial* industries, which did show associations with fulfillment.

The *Office* industry may share similarities with *Public administration, education and health care* and often consists of 100+-employee enterprises, which could explain the missing association between *Office* and fulfillment of ATP.

The ATP addresses the issue that the WEA generally has given few injunctions in the areas of psychosocial and ergonomic working environment [20].

The majority of injunctions have been issued in relation to accident risks, machine safety, and chemical exposures [20]. This is despite the fact that there are comprehensive human and economic expenses associated with both psychosocial and ergonomic working environment problems [[21], [22], [23], [24]].

The ATP provides the labor inspectors the opportunity of addressing the psychosocial and ergonomic OSH problems that otherwise would require the extensive work and documentation related to an injunction. Still, most enterprises receive the most ATPs in the areas where they typically receive the most injunctions; for example, most offices are more likely to deal with psychosocial issues than ergonomic-, indoor climate–, and chemistry-related issues, and the majority of their ATPs regarded the psychosocial working environment and not the ergonomic one.

Enterprises may have rejected ATPs regarding the ergonomic working environment, particularly large enterprises, if they perceived the solution to the ATP to be simpler, more technical, or less demanding than most psychosocial ATPs, which are often more complex. For example, if the ATP regarded a suspicion of a bakery's mixing bowls being too heavy for the staff, it would be relatively easy to replace the bowls with smaller ones. In other words, the enterprises may be willing to risk an injunction and solve the ergonomic issue without having to comply with the administration and obligations that come with an ATP.

Some larger enterprises may receive more inspections from the WEA than smaller ones, and therefore, these might know the WEA and their procedures better than other and smaller enterprises would. Previous studies have shown that labor inspectors who show support and offer the enterprise autonomy increase the enterprises’ compliance with law [25]. However, research regarding the effectiveness of these dialog-based inspections practices is scarce [12]. Interviews stemming from this evaluation have shown that some, and especially large, enterprises have good professional relationships with the labor inspectors and therefore might be more motivated and inclined to accept the ATP and benefit from the guidance provided by the WEA.

A smaller share of microenterprises (<10 employees) fulfilled an agreement than large enterprises. A study from 2020 found that inspectors identified a need for increased competence about working environment issues in microenterprises [26], which may explain why some of the microenterprises welcomed the ATP and some enterprises still lacked the competence and resources to accept the agreement and take the necessary steps to fulfill the agreement. Small enterprises lack in-house OSH expertise and favor pragmatic, low-cost solutions. A 2024 scoping review of successful OSH strategies in small- and medium-sized enterprises found that a “participatory and collaborative approach” is one of four core success factors for effective OSH management in small firms [10]. These factors play an important role in the ATP, and as such, the small enterprises should benefit greatly from the ATP compared to injunctions. Even so, it seems that there are barriers for small enterprises when it comes to accepting and fulfilling an ATP agreement.

An analysis from 2011 about special inspections showed that enterprises in the industry *Public administration, education and health care* expressed satisfaction with the guidance and dialog they received and that these inspections were constructive regarding the working environment [27].

Traditionally, *Construction, manufacturing, agriculture,* *and forestry* are considered “high risk.” In 2018, 20.5% of all fatal accidents in the 27 countries in the European Union took place in construction [4], and ATP seems to address these accident risks seeing as 80% of ATPs regarding accident risk are fulfilled.

A drastic change of perspective and culture is needed to truly assure a collective, participatory, bottom-up mindset positioning the employee in low-skilled jobs in the center [28], e.g., jobs in *Industrial, raw materials, supply* and *Trade, transportation etc.* The ATP agreements seem to reach these two types of industries as they make up 45% of the total number of ATP's across industries.

The construction industry entails a large part of mobile worksites, and a study from 2017 concluded that subcontracting means that labor inspectors must secure information from site managers and crews on the spot. Practical coaching during a walk-around often works better than written orders alone [9]. This coincides with the 72% of the enterprises in *Industrial, raw materials, supply* that fulfilled an ATP. Enterprises in this industry may find a dialog-based inspection practice to be more effective in improving competence and safety and health.

Seen from the managers' perspective, dialog-based workplace interventions and support from a rehabilitation coordinator can strengthen their competence and ability to act in complex return-to-work processes [29]. Psychosocial problems and agreements—which can be very complex—were fulfilled more than other problems and ATP may be a constructive inspection practice in regards to psychosocial OSH problems.

This dialog-based inspection practice showed promising results in addressing and solving psychosocial and ergonomic OSH problems, which typically are complex and time-consuming to uncover and document for labor inspectors. Across all enterprises, there was a large share of fulfillment of the ATP. The enterprises' industry, the number of employees, and type of OSH problem were associated with fulfillment of the ATP. Small enterprises and enterprises in the private sector received more ATPs regarding ergonomics than large and public enterprises, which mostly received psychosocial ATPs.

Small enterprises with fewer than 35 employees are less likely to fulfill an agreement than enterprises with 35 or more employees. Compared to ATPs regarding the psychosocial OSH, ergonomic, noise, and indoor climate ATPs had a lower probability of fulfilling the ATP.

Moving forward, the ATP initiative or similar initiatives may pay added attention to small enterprises and ATPs regarding ergonomic working environment in order to increase the fulfillment of ATP and improving the working environment for various types of enterprises.

Further research is needed in order to investigate the effectiveness of this type of initiative and inspection practice on various outcomes in the working environment.

### 4.1 Strengths and limitations

To our knowledge, this is the first study to investigate the association between enterprise characteristics and the compliance with this type of dialog-based inspection practice aimed at psychosocial and ergonomic OSH problems.

The data in this study stemmed from national registers, and we used updated information of all ATPs in the years 2020–2023. The large sample size (n = 3183) minimizes the risk of type 2 error. The implementation of ATP was a nation-wide initiative, so all enterprises with a labor inspection had the same predisposition to ATP offers, minimizing the selection bias.

This study was a quasi-experiment without controlled environments and clear randomization. We assumed an as-if randomization [30] since there was little or no difference in fulfillment of ATPs between the year of offer (apart from 2023) and the three supervision centers covering all Danish enterprises.

When the WEA implemented ATP as a reaction form, there were several other new initiatives implemented by the WEA, possibly causing confusion and resistance against this type of reaction among the enterprises as well as inspectors in the early years of implementation.

The analyses in this study did not include data on individual factors, e.g., life style, education, or the enterprises' organizational and economic situation. We did not have information about the reasons for not fulfilling the ATP, but the analyses indicate that ATPs in small enterprises and ATPs regarding ergonomics are less likely to have fulfilled ATP than large enterprises and psychosocial ATPs.

The smallest groups of industries, *Culture, leisure, other services* and *Office*, showed no associations with fulfillment of ATP. *Culture, leisure, other services* included 143 enterprises, and *Office* included 221 enterprises. The variation in group-size across industries may explain the missing statistical association. Another explanation could be that enterprises in *Public administration, education and health care*, *Culture, leisure, other services,* and *Offic*e are likely to have similar jobs and workflows and therefore, fulfilled roughly the same proportion of ATPs.

The registered number of employees in the national register is a self-reported measure and is susceptible to mistakes or nonupdated numbers.

There are benefits of a dialog-based approach to OSH, but it is not always possible to determine exact impacts on working conditions [11], and we were not able, in our study, to determine effects of the ATP on factors in the working environment or sickness absence.

Future research could benefit from studies investigating the long-term effect of fulfilling an ATP or other dialog-based inspection practices on the working environment and OSH. This was not possible in this study because of the study design; there were no control groups or baseline and follow-up measurements, making it impossible to know whether the ATP affects, for example, the number of accidents or the sickness absence in the enterprises.

## CRediT authorship contribution statement

**Mikala E. Jakobsen:** Writing – original draft, Methodology, Formal analysis, Data curation, Conceptualization. **Asta Kjærgaard:** Writing – review & editing, Validation, Investigation. **Emilie M. Rudolf:** Writing – review & editing, Validation, Methodology, Investigation. **Julie Palmqvist:** Writing – review & editing, Validation, Methodology, Investigation. **Jeppe Z.N. Ajslev:** Writing – review & editing, Validation, Project administration, Funding acquisition, Conceptualization.

## Conflicts of interest

Authors declare no conflicts of interest.
